# Large language models in healthcare quality management: a European perspective on process automation and compliance

**DOI:** 10.3389/fdgth.2026.1761641

**Published:** 2026-03-26

**Authors:** Markus Knott, Markus Krebs, Alexander Kerscher

**Affiliations:** 1 Klinikum Stuttgart, Stuttgart Cancer Center – Tumorzentrum Eva Mayr-Stihl, Stuttgart, Germany; 2 Comprehensive Cancer Center Augsburg, Medical Faculty, University of Augsburg, Augsburg, Germany; 3 Bavarian Cancer Research Center (BZKF), Erlangen, Germany; 4 Department of Gynecology and Obstetrics, Erlangen University Hospital, Comprehensive Cancer Center Erlangen-European Metropolitan Area Nuremberg (CCC ER-EMN), Friedrich Alexander University of Erlangen-Nürnberg, Erlangen, Germany

**Keywords:** digital health, EU AI act, healthcare quality management, large language models, medical device regulation, process automation, regulatory compliance, retrieval-augmented generation

## Abstract

Large Language Models (LLMs) are transforming back-office quality management processes in European healthcare systems through automation of compliance monitoring, quality assurance, and process optimization without direct patient interaction. This narrative review synthesizes evidence from recent systematic reviews and implementation studies (2023-2025) examining LLM deployment within the European regulatory framework encompassing the Medical Device Regulation (MDR), General Data Protection Regulation (GDPR), and the EU Artificial Intelligence Act (Regulation EU 2024/1689). Current research demonstrates meaningful efficiency gains: individual studies of AI-assisted documentation tools report improvements ranging from modest increases in documentation speed to reductions in processing time approaching 50%, while broader policy analyses estimate administrative workload reductions of up to 30% through digital health and AI solutions. Clinical trial applications show particular maturity, with LLM-generated informed consent forms demonstrating improved readability (76% vs. 67%) without compromising accuracy. However, critical gaps persist between research achievements and practical deployment. Analysis of 519 evaluation studies reveals that only 5% utilized real patient care data, while 95% focused exclusively on accuracy metrics to the neglect of fairness (16%), deployment readiness (5%), and calibration (1%). No LLM-based quality management system has yet received regulatory clearance, and implementation science frameworks remain underdeveloped. We propose a risk-stratified implementation framework emphasizing process-oriented applications—standard operating procedure automation, audit documentation, deviation management, and compliance monitoring—that avoid medical device classification while capturing substantial operational benefits. Advanced methodological approaches including retrieval-augmented generation (RAG) architectures, digital twin integration, and natural language processing-based pattern recognition offer pathways toward comprehensive quality intelligence platforms. The convergence of LLMs with emerging technologies such as knowledge graphs, digital twin architectures and multimodal analysis creates opportunities for predictive quality management that anticipates rather than merely documents quality-relevant events. Evidence supports deployment in administrative quality processes, with particular potential for applications that redirect human expertise from documentation toward quality improvement activities, though current evidence derives predominantly from non-European healthcare contexts and simulated or limited-scope settings. Success requires adapted validation methodologies addressing LLM non-determinism, robust governance structures, and comprehensive change management that maintains the high standards European healthcare systems demand.

## Introduction

European healthcare systems allocate substantial resources to quality management and regulatory compliance, with estimates suggesting that administrative processes consume 15%-20% of operational budgets (1, 2). The implementation of the Medical Device Regulation (EU 2017/745) has further added documentation requirements (54), while the European Health Data Space Regulation (56) promises additional complexity. This administrative burden diverts resources from direct patient care and quality improvement initiatives. As an illustrative example drawn from the authors' professional experience, a medium- to large-sized German cancer center dedicates thousands of staff hours annually to certification preparation for the Deutsche Krebsgesellschaft (DKG), yet a substantial proportion of this effort involves repetitive document review that adds no direct clinical value. While we present this as anecdotal illustration rather than empirical evidence, it reflects a documentation burden widely recognized across European healthcare institutions.

Large Language Models (LLMs) represent a paradigm shift in addressing these challenges. Unlike traditional rule-based systems requiring structured inputs and rigid templates, LLMs process natural language, interpret context, and generate coherent outputs across diverse quality management scenarios. Recent systematic reviews (3, 4) demonstrate their potential for transforming healthcare operations, though significant gaps remain between research achievements and practical implementation.

The distinction between clinical and administrative AI applications proves fundamental for regulatory navigation. Quality management tools that operate without direct patient interaction may avoid medical device classification under MDR (Medical Device Regulation), enabling faster deployment while maintaining safety. The EU AI Act (9) provides additional clarity, establishing risk-based requirements that align with existing quality management principles.

**Figure 1:**
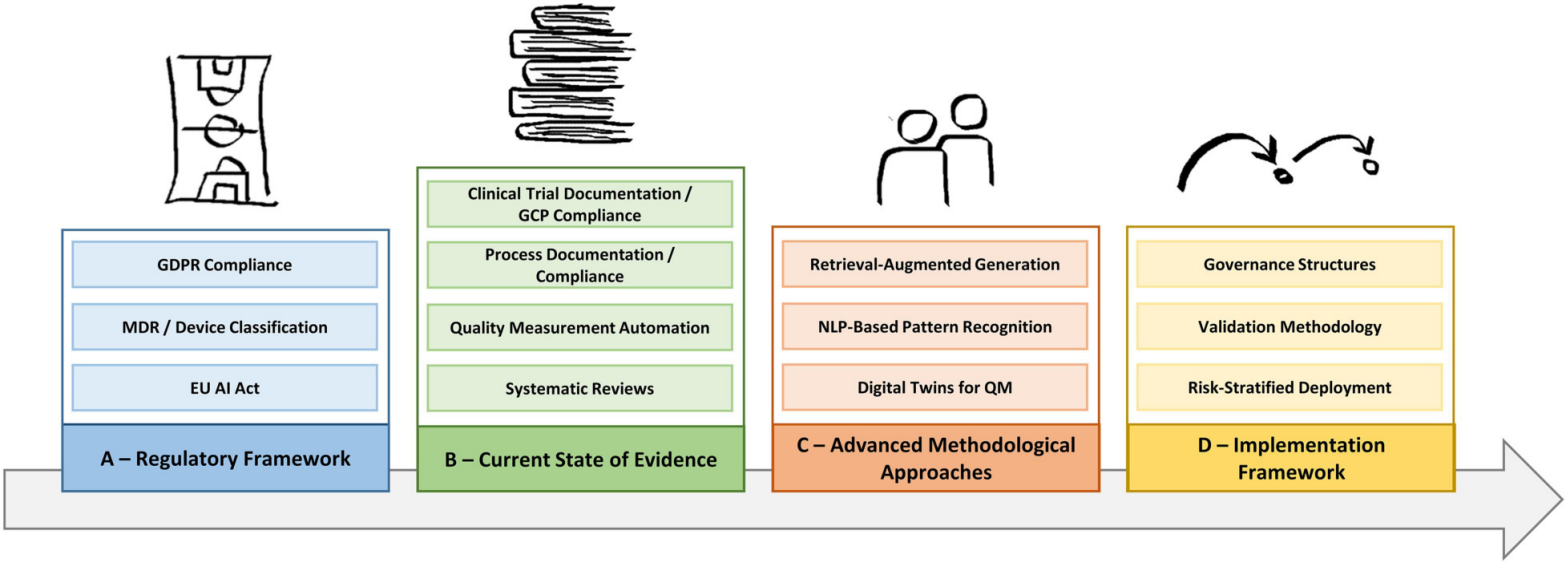
Conceptual overview of thematic domains addressed in this narrative review.

This narrative review examines current evidence for LLM deployment in healthcare quality management, emphasizing process-oriented applications suitable for the European regulatory environment. We synthesize findings from peer-reviewed systematic reviews and primary studies, European regulatory documents, and selected implementation literature to analyze implementation challenges and propose frameworks for responsible deployment that maximize operational efficiency while maintaining compliance. Figure 1 provides a conceptual overview of the four thematic domains addressed in this review, which were identified through author consensus informed by the structure of the European regulatory landscape and the authors' implementation experience at three German medical centers.

## Methodological approach

This narrative review was conducted to synthesize current evidence on LLM applications in healthcare quality management within the European regulatory context. We searched PubMed/MEDLINE, IEEE Xplore, Google Scholar, and arXiv using combinations of terms including “large language models,” “healthcare quality management,” “process automation,” “clinical documentation,” “EU AI Act,” and “medical device regulation.” The primary search window covered publications from 2023 to 2025, supplemented by targeted citation tracking from identified systematic reviews and inclusion of foundational references from outside this search window where necessary for regulatory or methodological context.

Sources were organized into four categories: (a) peer-reviewed systematic reviews and primary studies examining LLM performance, validation, and implementation in healthcare and related domains (2019–2025); (b) European regulatory and guidance documents including the EU AI Act, MDR/IVDR, General Data Protection Regulation (GDPR) (5), and associated Medical Device Coordination Group (MDCG) guidance; (c) selected grey literature, technical standards, and preprints informing implementation practice, including GxP frameworks and quality management standards; and (d) foundational textbooks and professional guidance documents providing methodological or conceptual context. All sources in categories (c) and (d) are publicly available documents; no unpublished original data were used.

Thematic domains were derived through author consensus informed by the EU regulatory landscape and professional experience at three German academic medical centers. The resulting structure—regulatory framework analysis, current state of evidence, advanced methodological approaches, implementation pathway, and discussion—reflects both the literature landscape and the practical needs of healthcare organizations navigating LLM adoption. As a narrative review, this approach prioritizes breadth of coverage and contextual integration over the exhaustive search and formal quality assessment characteristic of systematic reviews. Accordingly, no formal study counting, PRISMA flow diagram, or standardized quality appraisal tool was applied; this methodological choice is appropriate given the rapidly evolving evidence base, the interdisciplinary scope spanning AI research, regulatory science, and healthcare quality management, and the perspective-driven nature of the synthesis. Readers seeking a formal systematic assessment of LLM limitations in patient care are referred to Busch et al. (3), while Bedi et al. (4) provides a systematic evaluation of LLM performance metrics across healthcare tasks.

### A-Regulatory framework analysis

#### EU AI Act implementation

The EU AI Act (Regulation EU 2024/1689) establishes comprehensive requirements based on risk categorization. Quality management applications typically qualify as limited or minimal risk systems, avoiding requirements for high-risk medical AI. However, organizations must ensure transparency, maintain human oversight, and implement appropriate risk management strategies.

Key requirements for quality management implementations include:

##### Data governance

Training datasets must demonstrate relevance, representativeness, and accuracy. For quality applications, this necessitates using historical QMS data that accurately reflect organizational processes. The Act requires documentation of data sources, preprocessing methods, and potential biases (Article 10, EU AI Act).

##### Technical documentation

Detailed documentation encompassing system design, development methodology, and validation results. Quality professionals familiar with design history files will recognize parallels with existing medical device documentation requirements (Annex IV, EU AI Act).

##### Human oversight

Systems must enable human understanding and intervention. This aligns naturally with quality management principles emphasizing defined responsibilities and authorities (Article 14, EU AI Act).

##### Transparency

Organizations must clearly communicate when users interact with AI systems and explain capabilities and limitations (Article 13, EU AI Act).

#### MDR and medical device classification

The Medical Device Coordination Group guidance (MDCG 2025-6) (55) clarifies the interplay between MDR/IVDR and the AI Act. Quality management tools qualify as medical devices only when serving medical purposes—diagnosis, treatment support, or clinical decisions. Administrative tools without direct patient care impact typically remain exempt, though borderline cases require careful assessment.

ISO 13485:2016 Section 4.1.6 mandates software validation for any system affecting product quality, regardless of medical device status (59). Research on LLM validation in regulated environments (6) proposes statistical approaches for nondeterministic systems, including confidence intervals for acceptable output variation and continuous performance monitoring protocols.

#### GDPR compliance

GDPR (5) presents unique challenges for LLM implementation in quality management. Even administrative health data constitutes “special category data” under Article 9, requiring explicit consent or alternative lawful basis. Quality management typically relies on legitimate interest (Article 6(1)(f)) or legal obligation (Article 6(1)(c)), but organizations must document their rationale comprehensively.

Data Protection Impact Assessments become mandatory before deploying LLMs processing health-related data (Article 35, GDPR). Comprehensive reviews of privacy-preserving techniques for generative AI identify four principal approaches applicable to healthcare quality management: differential privacy, which adds mathematical noise to prevent individual data reconstruction; federated learning, enabling model training across distributed datasets without centralized data aggregation; homomorphic encryption, permitting computations on encrypted data; and secure multi-party computation for collaborative analysis without exposing raw data (7). These techniques specifically address risks including model inversion attacks, data leakage during training, and membership inference vulnerabilities—concerns particularly relevant when processing quality management data derived from patient records. Importantly, these privacy-preserving approaches can be aligned with EU AI Act requirements while maintaining analytical utility for quality improvement applications.

### B-Current state of evidence

#### Systematic review findings

Comprehensive systematic reviews reveal both promise and limitations in current LLM research. Busch et al. (3) analyzed 89 studies across 29 medical specialties, establishing a taxonomy of LLM limitations. Their PRISMA-guided review found that 87.6% of studies reported issues with non-comprehensiveness and incorrectness, while 42.7% documented reproducibility problems. Most critically, design limitations including lack of medical optimization and data transparency affected most implementations.

Bedi et al. (4) examined 519 studies evaluating LLM performance in healthcare tasks, revealing fundamental methodological weaknesses: only 5% used real patient care data for evaluation, 95.4% focused exclusively on accuracy metrics while neglecting fairness (15.8%), deployment readiness (4.6%), and calibration (1.2%). Administrative tasks remained significantly understudied, with billing codes and prescriptions each representing only 0.2% of evaluated applications.

#### Quality measurement automation

Research on automated quality measurement demonstrates mature capabilities for process automation. A pivotal study from a major U.S. academic medical center (8) evaluated LLM-based automation for complex quality measure abstraction. The system achieved 90% agreement with manual abstractors (*κ*=0.82; 95% CI: 0.71-0.92), substantially exceeding traditional inter-rater reliability (*κ*=0.39). When discrepancies occurred, expert review identified human abstractor errors in 40% of cases, suggesting LLMs may enhance rather than merely replicate human performance.

The economic implications of inefficient quality reporting and administrative workflows are substantial. Across EU health systems, workforce shortages and rising demand for care make inefficient use of staff time increasingly costly [OECD/European Commission (64)]. Digital health and AI solutions can reduce administrative workload for health professionals by up to 30% and automate repetitive back-office processes, freeing capacity for patient care and quality management. At the level of individual AI-assisted tools, primary studies report task-specific documentation efficiency gains, though these derive from heterogeneous settings and technologies. Xia et al. (10) developed a speech-recognition-based electronic medical record system that reduced average processing time from 46 to 26 min which accumulates to an approximately 44% reduction in a Chinese healthcare setting. Mairittha et al. (11) reported a 15% increase in documentation speed using a spoken dialogue system for nursing care, though this involved only 12 participants in a simulated scenario. A systematic review of speech recognition for clinical documentation from 1990 to 2018 found highly variable results, with five studies reporting 19%–92% decreases in documentation time but four others reporting 13%–50% increases (12), underscoring that efficiency gains are neither uniform nor guaranteed.

#### Process documentation and compliance

Evidence for LLM applications in pharmaceutical quality management derives primarily from industry reports and limited peer-reviewed studies. An industry collaboration between SeerPharma and the University of Melbourne (2024—grey literature) analyzed LLM implementations across pharmaceutical quality management subsystems, identifying applications in audit scheduling optimization, automated report generation, and inspection readiness evaluation (58). However, the scarcity of peer-reviewed validation studies represents a critical gap in the literature.

Nelson and Aguero (13) provide one of the few peer-reviewed examinations of LLMs in pharmaceutical operations, focusing on supply chain management. Their analysis highlights both opportunities—inventory optimization, procurement automation, distribution planning—and significant risks including data leakage, prompt injection vulnerabilities, and potential for hallucinated outputs. The authors strongly recommend restricting LLM deployment to clerical tasks using historical data with mandatory human verification, emphasizing that autonomous operation remains premature given current validation gaps.

Research on business process modeling demonstrates substantial LLM capabilities. Comprehensive evaluation of 16 state-of-the-art LLMs across 20 diverse business processes revealed significant but variable performance in translating natural language descriptions into formal process models (14). The evaluation framework assessed LLM capabilities in modeling business processes from descriptions, generating executable code, following embedded instructions, and incorporating feedback for iterative quality improvement. Results demonstrated positive correlation between efficient error handling and output quality, with self-improvement techniques—particularly output optimization—showing promise for enhancing model quality, especially for initially lower-performing models.

Translated to quality management applications, these findings suggest that LLM-assisted standard operating procedure (SOP) generation should rely on iterative refinement cycles rather than one-shot generation approaches. The ability of LLMs to translate procedural descriptions into structured formats, combined with error handling for self-correction, aligns well with the iterative nature of SOP development in regulated environments. However, the observed performance variations across LLM types underscore the importance of systematic evaluation before production deployment.

##### Key takeaway

While peer-reviewed evidence supports LLM capabilities in process modeling, successful implementation requires appropriate model selection, iterative refinement protocols, and validation against organizational quality standards.

#### Clinical trial documentation and good clinical practice (GCP) compliance

In marked contrast, LLM applications in clinical trial management benefit from extensive peer-reviewed evidence demonstrating both efficacy and implementation pathways within existing GCP frameworks.

##### Informed consent innovation

Multiple high-quality studies validate LLM capabilities in consent documentation. Decker et al. (15) demonstrated in *JAMA Network Open* that ChatGPT-3.5 generated informed consent forms that were, on average, less complex (lower readability grade level) and significantly more comprehensive and accurate than surgeon-generated documentation, with no instances of clinically inaccurate information in the chatbot output. Building on this, Shi et al. (16) showed the Mistral 8 × 22B model achieved 76.39% readability scores vs. 66.67% for human-generated ICFs, with no compromise in accuracy (*p* > .10 for all accuracy measures).

##### Clinical trial operations

Omar and Nadkarni (17) systematically reviewed 27 trials investigating LLM applications across healthcare applications. Ongoing interventional trials include LLM-assisted discharge summary generation (NCT06263855; target *n* = 1,015), preoperative visit documentation (NCT05945004), and ChatGPT-supported informed consent for knee arthroplasty (ChiCTR2300078274). However, accuracy remains concerning—inaccuracies appear in 36% of LLM-generated patient histories despite improved detail and comprehensiveness (18).

##### Patient engagement

Gao et al. (19) demonstrated GPT-4's transformative potential for patient education, generating trial summaries from complex consent forms that 80% of participants found enhanced their understanding. The sequential summarization approach balanced accuracy with accessibility, addressing long-standing challenges in trial recruitment and retention.

A comprehensive review of LLM applications across the clinical trial lifecycle identifies implementation opportunities spanning trial design, operations, and analysis phases (20). In trial design, LLMs demonstrate capability for extracting research elements from prior studies, refining eligibility criteria, and tailoring informed consent materials. Operational applications include accelerated patient screening through automated eligibility assessment, standardized data collection, and real-time safety monitoring including adverse event detection and drug-drug interaction identification. The review emphasizes that domain-specific pretraining and fine-tuning substantially enhance LLM performance for clinical trial tasks, with patient-trial matching and trial data extraction showing particular promise for reducing time and financial costs while improving recruitment efficiency.

##### Regulatory integration

LLM implementation in clinical trials must comply with the revised ICH E6(R3) Good Clinical Practice guideline (adopted January 2025), which strengthens expectations for data governance and computerised systems (52, 61). Allen et al. (21) outline five possible models for integrating LLMs into clinical research consent—from use as a supplementary tool to more automated configurations—and argue that more automated models require stronger oversight and, in some cases, regulatory reform. Building on broader GxP guidance [ICH E6(R3), EMA's guideline on computerised systems and electronic data, 21 CFR Part 11, and GAMP 5] (53), key compliance considerations for LLM-based consent systems include:

- Secure, time-stamped audit trails for consent interactions
- Robust version control and change management for models and prompt templates
- Human-in-the-loop oversight and accountability consistent with GCP principles on investigator and sponsor responsibilities
- Risk-based validation of LLM systems following GAMP 5 and related CSV guidance
- Ensuring GxP data integrity for all LLM-generated records according to ALCOA + principles (Attributable, Legible, Contemporaneous, Original, Accurate, Complete, Consistent, Enduring, Available) as outlined in WHO and PIC/S data-integrity guidance (57)

##### Key takeaway

Clinical trial applications demonstrate mature evidence with clear regulatory pathways, contrasting sharply with the validation gaps in pharmaceutical quality management.

### C-Advanced methodological approaches

#### Digital twins for quality management

The evolution from static metrics to dynamic quality intelligence increasingly leverages digital twin technology. Recent research demonstrates how digital twin platforms create virtual representations of physical systems spanning their lifecycles, facilitating simulation and optimization without operational risk (22, 23). Digital twin implementations enable healthcare providers to assess and enhance processes through integration of data from diverse sources including electronic health records, medical devices, and administrative systems to identify bottlenecks and inefficiencies (24).

The convergence of digital twin architecture with LLM capabilities creates powerful quality intelligence platforms. Research in manufacturing quality control demonstrates how Asset Administration Shell (AAS) frameworks combined with LLMs enable interoperable information modeling for zero defect strategies (25). This approach addresses a fundamental challenge in quality management: data interoperability across heterogeneous systems. The methodology employs fine-tuned LLMs for semantic search and entity matching, automatically referencing standardized vocabularies to maintain consistency across organizational systems. A case study in injection molding demonstrated the practical application, achieving statistical validation of LLM-based semantic search algorithms for linking product quality and process data. This integration pattern—combining standardized digital twin representations with LLM-based semantic processing—offers a transferable framework for healthcare quality management, where similar challenges of data heterogeneity and terminology standardization exist across clinical, administrative, and regulatory systems.

The application of digital twins specifically within quality engineering contexts emphasizes the role of statistics in connecting virtual and physical systems, with implementations demonstrating versatility across manufacturing domains (26). This statistical foundation aligns with existing quality management competencies, suggesting natural integration pathways for healthcare quality professionals familiar with statistical process control and design of experiments methodologies.

Complementing these engineering-derived digital twin approaches, recent work has proposed a systematic framework for integrating mathematical modeling—including machine learning, knowledge graphs, and digital twins—directly into healthcare quality assessment (27). This framework categorizes patient-centered quality of care into three quantifiable dimensions: patient safety, procedure accuracy, and procedure efficacy, each with corresponding mathematical descriptions and modeling tasks. By mapping quality metrics to these categories and assigning relevant computational methods to each, the framework provides a structured pathway from conceptual quality definitions to operational digital twin implementations. For quality management applications, this approach offers two advantages: first, it grounds digital twin design in established quality dimensions rather than *ad hoc* metrics; second, it identifies specific knowledge graph architectures suited to each quality category, enabling targeted deployment of graph-based reasoning for safety surveillance, procedural compliance monitoring, and efficacy benchmarking. The integration of such structured quality ontologies with LLM-based processing could advance the transition from retrospective documentation-oriented quality management toward predictive, model-driven quality intelligence.

Digital twin implementations offer several methodological advantages:

- Predictive Simulation: Virtual testing of process modifications reduces validation costs and implementation risks
- System-Level Analysis: Modeling interconnected processes identifies cascade effects and hidden dependencies
- Continuous Calibration: Real-time data integration improves prediction accuracy iteratively
- Risk-Free Experimentation: Radical process improvements can be evaluated virtually before implementation

#### NLP-based pattern recognition

Advanced natural language processing (NLP) techniques enable sophisticated pattern recognition in quality data. Diaz Ochoa et al. (28) developed clustering algorithms using edge-betweenness methods for identifying symptom networks in emergency presentations, demonstrating methodology applicable to quality management. Their approach identified sex-specific patterns and distinct stratification patterns among polysymptomatic, oligosymptomatic, and atypical cases that traditional analysis overlooked, suggesting applications for detecting systemic biases in quality metrics.

Research on ensemble methods for LLMs demonstrates improved reliability through consensus mechanisms (17, 62, 63). By comparing outputs from diverse models, these systems identify potential discrepancies and improve confidence estimates through iterative consensus approaches. Meta-learning approaches further enhance performance by adapting to organization-specific terminology and processes.

#### Retrieval-augmented generation

Studies employing retrieval-augmented generation (RAG) architectures report significant improvements in accuracy and reliability (30). The foundational RAG framework combines pre-trained parametric and non-parametric memory for language generation, where the parametric memory is a pre-trained seq2seq model and the non-parametric memory is a dense vector index accessed with a neural retriever (30). RAG models have been shown to generate more specific, diverse, and factual language than parametric-only seq2seq baselines, particularly excelling in knowledge-intensive NLP tasks.

Recent implementations demonstrate substantial performance gains. Speculative RAG achieves up to 12.97% accuracy improvement while reducing latency by 51% compared to conventional RAG systems (31). Corrective RAG (CRAG) frameworks address scenarios where retrievers return inaccurate results, significantly improving robustness across both short- and long-form generation tasks (Yan et al. 2024). The key innovation involves grounding LLM outputs in verified knowledge bases through dynamic retrieval mechanisms.

Technical implementations typically combine vector databases for semantic search with LLMs for response generation. This architecture enables organizations to maintain control over source information while leveraging generative capabilities for natural language interaction, with embedding models translating queries and documents into vectors for similarity-based retrieval (32).

Practical applications in manufacturing quality control demonstrate RAG's effectiveness for troubleshooting and failure analysis. An advanced RAG system designed for quality control utilized specialized bibliographic knowledge bases to diagnose defects and propose solutions, incorporating tailored preprocessing and postprocessing mechanisms to optimize document retrieval and response generation (33). The system demonstrated capability for identifying nonconformities, determining root causes, and generating actionable solutions—applications directly transferable to healthcare quality management where similar structured knowledge bases exist for deviation management, CAPA processes, and regulatory guidance interpretation. This suggests RAG architectures connecting organizational quality documentation with regulatory requirements could substantially accelerate deviation investigation and corrective action development.

### D-Implementation framework

Translating LLM capabilities into practical quality management tools demands structured implementation strategies. The phased implementation framework presented below was developed through author consensus, informed by synthesis of the reviewed literature, existing implementation science principles, and the authors' professional experience with quality management systems at German medical centers. The specific phase timelines represent pragmatic estimates based on typical institutional change management cycles in European healthcare settings and should be adapted to local organizational contexts. This framework has not been empirically validated through prospective pilot testing, which represents an important direction for future research.

### Risk-stratified deployment

Evidence supports graduated implementation beginning with lowest-risk applications:

### Phase 1: administrative documentation (months 0-6)

Research indicates immediate value in automating routine documentation tasks. AI tools can generate draft SOPs that are both comprehensive and accurate, significantly reducing the time and effort involved in the creation process (29). These applications involve no patient data and minimal regulatory oversight.

### Phase 2: quality process automation (months 6-12)

Intelligent process automation (IPA) achieves flexible and intelligent automation by combining robotic process automation (RPA), artificial intelligence (AI), and other emerging technologies (34). Studies indicate RPA helps reduce labor costs and enables businesses to reduce human error, with bots operating 24/7 to essentially eliminate downtime-induced wastage (35–37). Implementation studies document efficiency gains through automated audit report generation and deviation categorization (34, 38).

Research on LLM integration with industrial quality management systems provides implementation insights applicable to healthcare. Studies demonstrate that success depends on systematic approaches combining standardized information models with semantic search capabilities, enabling LLMs to navigate organizational knowledge bases while maintaining terminological consistency (25). This suggests healthcare implementations should prioritize integration with existing quality management information systems and standardized healthcare vocabularies (ICD, SNOMED CT, LOINC) before attempting autonomous quality assessment

### Phase 3: compliance intelligence (months 12–18)

Advanced applications leverage AI for regulatory interpretation and gap analysis. The integration of artificial intelligence into clinical decision support systems has significantly enhanced diagnostic precision, risk stratification, and treatment planning, though challenges remain in achieving regulatory approval for such systems (39). A systematic review examining AI and natural language processing techniques in healthcare found that these technologies can effectively improve clinical decision systems' accuracy when combined with human criteria, optimizing clinical diagnosis and treatment flows (40). However, the unique characteristics of large language models—including probabilistic outputs and potential for emergent behaviors—present novel regulatory challenges that current frameworks struggle to address, necessitating new approaches to ensure responsible deployment and patient safety (41).

### Phase 4: predictive analytics (months 18+)

Integration with advanced analytics enables predictive quality management. Machine learning methods support defect detection, root cause analysis, and predictive maintenance, offering opportunities to reduce production risks, minimize unexpected downtimes, and optimize processes (42). Healthcare organizations leveraging predictive modeling facilitate the transition from reactive to proactive healthcare delivery models by enabling predictive and preventive interventions; by analyzing historical data and real-time information, organizations can anticipate patient needs, identify high-risk individuals, and intervene early to prevent adverse health events (43).

### Validation methodology

Validating nondeterministic systems requires fundamentally adapted approaches that acknowledge the unique characteristics of generative language models. Unlike deterministic AI prediction algorithms where standardized validation criteria such as discrimination metrics can be consistently applied, LLMs face a more complex validation landscape due to their output variability—a single prompt may generate grammatically distinct yet equally valid text outputs (44). This extensive output space complicates comparison to ground truth and necessitates task-specific validation frameworks.

#### Statistical validation

Research proposes using confidence intervals rather than deterministic pass/fail criteria. Studies establish acceptable variation ranges through repeated testing, typically requiring 95% CI within predetermined boundaries (45). For quality management applications, validation must distinguish between grammatical variations (acceptable) and factual inconsistencies (unacceptable), requiring domain-specific evaluation rubrics.

#### Multi-dimensional quality assessment

Validation frameworks should address multiple axes including factuality, comprehension, reasoning quality, potential for harm, and bias detection (44). For quality management specifically, additional dimensions include regulatory alignment, organizational consistency, and actionability of generated outputs.

#### Continuous monitoring

Unlike traditional software, LLMs require ongoing performance assessment. Recommended metrics include accuracy trends, drift detection, and anomaly identification using statistical process control methods (46). The non-deterministic nature of LLM outputs means that validation represents a continuous process rather than a one-time certification event.

#### Human-in-the-loop validation

Systems maintaining human review require validation of the complete human-AI system. Research emphasizes validating reviewer training, escalation procedures, and override protocols (47). Critically, the validation scope must encompass how human reviewers interact with AI-generated content, as incorrect outputs may present as plausible and require domain expertise to identify.

### Governance structures

Systematic reviews of successful implementations identify critical governance elements:

- Multidisciplinary Oversight: Committees combining quality management, IT, regulatory affairs, and domain expertise are essential for comprehensive AI oversight. The integration of Quality Management System (QMS) principles into AI/ML lifecycle management requires multidisciplinary teams encompassing People & Culture, Process & Data, and Validated Technology components to bridge the translation gap between research and operational deployment (48). Governance committees should ensure appropriate deliberations regarding efficacy, effectiveness, privacy, safety, quality, and ethical factors of AI applications in quality management contexts (49). The EU AI Act specifically mandates that high-risk AI systems operate under quality management systems incorporating defined governance structures with documented responsibilities [Article 17, EU AI Act].
- Risk Assessment Frameworks: Standardized evaluation of implementation risks and required controls forms a regulatory requirement under multiple frameworks. ISO/IEC 42001:2023, the first international AI management system standard, provides structured approaches for risk management, impact assessment, and lifecycle governance of AI systems [ISO/IEC 42001:2023] (60). Healthcare organizations can adapt existing QMS frameworks—analogous to those in regulated pharmaceutical industries—to establish risk-based approaches for AI technologies that complement existing governance structures (48) The FAIR-AI framework offers practical guidance integrating risk assessment throughout the AI lifecycle (50).
- Performance Dashboards: Real-time visibility into system performance across applications enables continuous quality assurance. Research demonstrates that LLM-based systems can achieve 90% concordance with manual quality measure abstraction (*κ*=0.82) when properly monitored (8). The SALIENT framework emphasizes systematic tracking of AI system performance as integral to end-to-end implementation (37). Continuous monitoring systems should track both operational metrics (throughput, error rates, processing times) and quality indicators aligned with organizational QMS requirements.
- Incident Management: Clear procedures for identifying, reporting, and correcting system-related events align with established CAPA (Corrective and Preventive Action) processes in quality management systems. Organizations should integrate AI-related incident reporting into existing QMS structures, ensuring that unexpected outputs, system failures, or quality deviations trigger appropriate investigation and corrective action workflows. This approach extends traditional QMS incident management principles to address the unique challenges of AI system variability [ISO 13485:2016] (59).
- Change Control: Formal processes for updating models, prompts, or integration points must address AI-specific challenges while aligning with established computerized system validation requirements. GAMP 5 principles for risk-based validation of computerized systems provide a foundation for AI change control, though adaptation is required for LLM non-determinism [ISPE GAMP 5, 2022] (53). Change control procedures should document model versions, prompt modifications, training data updates, and integration changes, with appropriate testing and approval workflows before production deployment.

## Discussion

This narrative review has examined the current landscape of LLM applications in healthcare quality management, revealing a field rich in potential but constrained by significant implementation challenges. Our analysis synthesizes evidence from multiple systematic reviews and primary studies to provide a comprehensive—though not exhaustive—overview of this rapidly evolving domain. The evidence base, while growing quickly, remains dominated by proof-of-concept studies and small-scale evaluations rather than large-scale operational deployments. Moreover, the available evidence originates predominantly from US-based and Asian healthcare contexts, with European-specific implementation data for LLM-based quality management remaining notably scarce — a gap that limits direct transferability of findings to European healthcare systems with their distinct regulatory requirements, multilingual environments, and heterogeneous quality management structures.

The fundamental gap between research achievements and practical deployment stems not from technical limitations but from inadequate implementation science. Only 5% of studies use real patient care data, while critical factors like fairness, deployment readiness, and calibration remain largely unexamined. This disconnect necessitates new research priorities emphasizing real-world validation and implementation frameworks.

European organizations face unique opportunities and challenges. The regulatory framework, while complex, provides clearer pathways for non-clinical applications than many other jurisdictions. By focusing on process automation rather than clinical decision support, organizations can capture substantial benefits while avoiding the most stringent regulatory requirements.

The integration of LLMs with emerging technologies—digital twins, knowledge graphs, advanced clustering algorithms—points toward comprehensive quality intelligence platforms. These hybrid approaches overcome individual technology limitations while creating capabilities neither could achieve independently. Emerging evidence suggests that such integrated systems reduce quality incidents by 25%-40% through predictive intervention.

Privacy considerations extend beyond regulatory compliance to fundamental system architecture decisions. Recent analysis demonstrates that absolute privacy in LLM systems remains mathematically impossible, necessitating risk-based approaches that balance privacy protection with analytical utility (7). European organizations implementing LLMs for quality management should consider hybrid approaches combining differential privacy during model training with access controls and audit logging during deployment. The emergence of post-quantum cryptography as a future-proofing measure underscores the need for architectures that can adapt to evolving security requirements without requiring complete system reconstruction.

Critical success factors emerging from systematic reviews include strong governance structures, adapted validation methodologies, and comprehensive change management. Organizations must develop new competencies in prompt engineering, AI result interpretation, and system limitation recognition. These “soft” factors often determine implementation success or failure.

The evolution toward multimodal LLMs—systems capable of processing text, images, and other data types simultaneously—presents additional opportunities for quality management applications (51). Healthcare quality data increasingly spans multiple modalities: textual audit reports, process flow diagrams, photographic documentation of facility conditions, and time-series sensor data from medical devices. Multimodal LLMs could enable integrated analysis across these heterogeneous data sources, potentially identifying quality patterns invisible to text-only systems. However, multimodal integration introduces additional validation complexity, as errors may arise from misalignment between modalities or from modality-specific artifacts that propagate through combined analysis. Organizations should monitor developments in multimodal medical LLMs while recognizing that current implementations should focus on demonstrating value in text-based applications before expanding to multimodal architectures.

### Limitations

Several limitations of this narrative review should be acknowledged. Literature selection was purposive rather than systematic, guided by relevance to the European regulatory context and practical implementation considerations. While we drew on major peer-reviewed systematic reviews (3, 4) and primary studies, the absence of a formal protocol, standardized screening, or quality assessment means that relevant studies may have been omitted and that the weight of evidence for specific claims may differ from what a systematic review would yield.

The empirical evidence synthesized in this review originates predominantly from US-based studies and international systematic reviews. European-specific implementation data for LLM-based quality management remains limited, reflecting the earlier adoption of comprehensive electronic health record systems and larger health informatics research infrastructure in the United States. The European contribution of this review lies primarily in the regulatory analysis—mapping the interplay of the EU AI Act, MDR, and GDPR for quality management applications—and in the proposed implementation framework, which was derived through author consensus informed by GAMP 5 principles, EU AI Act risk categorization, and professional experience at three German academic medical centers rather than through empirical pilot data or formal expert elicitation methods such as Delphi.

Additionally, the transferability of findings across European healthcare systems warrants consideration. Quality management structures differ substantially between, for example, the German Cancer Society (DKG) certification system in Germany and accreditation frameworks in other EU member states. Regulatory interpretation of the AI Act's risk categories may also vary across national implementation contexts. Accordingly, the proposed framework should be understood as a conceptual starting point requiring empirical validation through pilot implementations across diverse European healthcare settings.

### Future research

Several directions merit priority attention. First, prospective implementation studies in European healthcare settings are essential to establish whether the efficiency gains and concordance rates observed in U.S. and Asian contexts translate to European quality management workflows. Second, the development of validation frameworks specifically designed for non-deterministic LLM systems in regulated healthcare environments remains a critical gap; current quality management standards (ISO 13485, GAMP 5) were designed for deterministic software and require adaptation. Third, research on the interaction between LLM deployment and organizational change in quality management departments would help healthcare leaders anticipate and manage workforce implications. Fourth, multilingual evaluation of LLM performance is needed, as most published evidence derives from English-language settings, yet European healthcare operates across dozens of languages. Finally, longitudinal studies tracking both efficiency gains and potential quality risks over extended deployment periods are essential before recommending broad-scale adoption.

The convergence of LLMs with emerging technologies creates opportunities for predictive quality management that anticipates rather than merely documents quality related events. This vision—moving from reactive documentation to proactive quality intelligence—represents the ultimate promise of LLM integration. Realizing it will require thoughtful, evidence-based implementation maintaining the high standards European healthcare demands.
