# Supplementary material for: Large language models in healthcare quality management: a European perspective on process automation and compliance
Source: Front Digit Health. 2026 Mar 26;8:1761641. doi: 10.3389/fdgth.2026.1761641 (PMC13062252; doi:10.3389/fdgth.2026.1761641)
Supplement: Supplementary file 1 [file Datasheet1.docx]

*Large Language Models in Healthcare Quality Management:*

*A European Perspective on Process Automation and Compliance*

Markus Knott, Markus Krebs, Alexander Kerscher

**Supplementary Material S1: AI Prompt for Manuscript Preparation**

In accordance with Frontiers guidelines on the use of generative AI technologies in manuscript preparation, we disclose the following prompt used with Claude Opus 4.5 (Anthropic, San Francisco, CA, USA) for language editing and formal manuscript optimization:

**AI PROMPT**

You are an academic writing assistant specializing in medical and

healthcare research manuscripts. Your task is to review and optimize

the attached manuscript for submission to a peer-reviewed journal.

**SCOPE OF ASSISTANCE:**

- Improve readability, clarity, and flow of the text

- Enhance sentence structure and paragraph transitions

- Correct grammar, spelling, and punctuation errors

- Ensure consistent terminology throughout the manuscript

- Optimize academic tone and formal scientific writing style

- Verify adherence to journal formatting guidelines

- Suggest improvements to abstract structure and clarity

**STRICT LIMITATIONS:**

- Do NOT alter, add, or remove any scientific content, claims,

or conclusions

- Do NOT modify, add, or delete any references or citations

- Do NOT change the meaning or interpretation of any statements

- Do NOT introduce new arguments or remove existing ones

- Do NOT alter any numerical data, statistics, or quantitative

findings

- Preserve all author-specific terminology and conceptual

frameworks

**OUTPUT REQUIREMENTS:**

- Provide tracked changes or clearly marked suggestions

- Explain substantive edits briefly when relevant

- Flag any unclear passages for author review without

rewriting content

- Maintain the authors' voice and scientific intent throughout

**Author Responsibility Statement:** All AI-generated suggestions were critically reviewed, verified for accuracy, and edited by the authors. The authors retain full responsibility for the scientific accuracy, integrity, and originality of all manuscript content. No scientific content, conclusions, or references were generated or modified by the AI tool.

**AI Tool Specifications**

**Tool:** Claude Opus 4.5

**Developer:** Anthropic, San Francisco, CA, USA

**Access Date:** December 2025

**Purpose:** Language editing, readability optimization, and formal manuscript preparation

**Scope:** Stylistic and grammatical improvements only; no modification of scientific content, data, or references
